# Sleep and Daytime Complaints During Manic and Depressive Episodes in Children and Adolescents With Bipolar Disorder

**DOI:** 10.3389/fpsyt.2019.01021

**Published:** 2020-01-23

**Authors:** Maria Cecilia Lopes, Miguel Angelo Boarati, Lee Fu-I

**Affiliations:** Child and Adolescent Affective Disorder Program (PRATA), Department and Institute of Psychiatry at University of Sao Paulo Medical School, Sao Paulo, Brazil

**Keywords:** sleep, nocturnal enuresis, bipolar disorder, children, adolescents

## Abstract

**Introduction:**

Depressive and manic episodes of bipolar disorder can interact with sleep complaints, followed by a worsened psychiatric condition. The aim of this study was to examine the interaction of sleep disorders with bipolar disorder in youths during depressive and manic episodes.

**Methods:**

The target population was children and adolescents drawn from the Children and Adolescents Affective Disorder Program. Clinical assessment for current psychiatric diagnosis was done by direct clinical interview, Diagnostic Interview for Children and Adolescents (DSM-IV), and best-estimated clinical consensus. We applied sleep questionnaires from which we obtained sleep and daytime complaints during manic and depressive episodes. All statistical tests of significance were done using 2-tailed tests with α = 0.05.

**Results:**

Participants in this study comprised 29 children (age = 10 ± 3 years, boys = 23) and 43 adolescents (age = 15 ± 2.4 years, boys = 30). Sleep complaints were observed in 66.4% of participants during manic episodes and 52.3% during depressive episodes. 37.9% of patients had sleep complaints in both episodes. Time in bed was longer during depressive episodes than manic episodes (p = 0.01). We found a high prevalence of nocturnal enuresis in depressive episodes in children and adolescents, which was statistically significant compared with manic episodes (p < 0.05). Unrested sleep was higher in adolescents in both episodes, and it was statistically significant during manic episodes (p < 0.05).

**Conclusion:**

According to our analyses, the minority of patients had sleep complaints in both episodes. Our data showed that nocturnal enuresis occurred more frequently during depressive than manic episodes. Further research is necessary to understand the implications of these data.

## Introduction

Sleep complaints are frequently described in bipolar disorder (BD). The adverse consequences of poor sleep on mood, motivation, and cognitive changes have been associated with negative functional consequences in individuals with BD (1–5). Sleep disturbance may contribute to a relapse in BD as a prodromal symptom, or warning signal that can appear before an episode of depression or mania (6–8).

Jackson et al. (2003) (9) reported that the majority of patients with BD (over 80%) were able to identify early symptoms, with sleep disturbance being the most common prodrome of mania and the sixth most common prodrome of depression. Some authors have described a monoaminergic syndrome that can explain both pathophysiology in depression and sleep complaint with noradrenergic hyperactivity; in relation to this syndrome, “withdrawal-induced cholinergic overdrive and the cholinergic-monoaminergic system” are the two most investigated and supported models (10). Sleep disturbance may contribute to maintenance of symptoms and impairment followed by poor memory for the positive domains or events in their lives (11). However, there are few data about sleep and symptomatic daytime behaviors in childhood BD. The aim of this study was to describe the presence of sleep and daytime complaints in children and adolescents with bipolar disorder.

## Methods

The target population was children and adolescents drawn from the Children and Adolescents Affective Disorder Program at the Institute of Psychiatry of the University of São Paulo, Brazil, from May 2008 to December 2010. The inclusion criteria: to be enrolled in this study, patients should fulfill DSM-IV (12) criteria for BD type I, II, and NOS, in the Affective Disorder Program at the Institute of Psychiatry of the University of São Paulo, Brazil, from May 2008 to December 2010. Our study sample comprised 72 children and adolescents, of both genders, aged 06 to 18. The clinical assessment for current psychiatric diagnosis was done by direct clinical interview by two senior psychiatrists (LFI and MB) applying the Diagnostic Interview for Children and Adolescents- DSM-IV version, and best-estimated clinical consensus.

The sleep patterns were obtained in the application of sleep scale by a group of psychologists with a senior sleep physician (MCL), and by the psychiatric clinical interview using structure scales to analyze sleep patterns comparing depressive and mania episodes. Our protocol applied sleep questionnaires according to the Bruni scale (1996) (13) from which we obtained information about their sleep and daytime complaints, during manic and depressive episodes. There were the monthly support in the BD treatment. The sleep analyses were made after the diagnosis was done. The sleep state in each mood episode was defined and the sleep questions were also scored according to the intensity of the each symptom in a rate from 1 to 5: 5 Always (daily); 4 Often (3 or 5 times per week), 3 Sometimes (once or twice per week), 2 Occasionally (once or twice per month or less), 1 Never. Our sample were patients in undergoing treatment using different medications. All statistical tests of significance were done using 2-tailed tests with α = 0.05.

### Standard Protocol Approvals, Registrations, Patient Consents

Written informed consent was signed by the parents after the approval by the Ethics Committee of the institution. The study was approved by the Ethics Committee of the Clinical Hospital of the University of São Paulo, according to the Declaration of Helsinki.

## Results

The participants in this study comprised 29 children (age = 10 ± 3 years old, boys = 23) and 43 adolescents (age = 15 ± 2.4 years old, boys = 30). Sleep complaints were observed in 78% of participants during manic episodes and 80.1% during depressive episode; 37.9% of participants had sleep complaints in both episodes.

The characteristics of the BD was described in the: **Table 1**. The occurrence of sleep complaints were observed and we found differences between manic versus depressive episodes in youth with BD (see **Table 2**). Interesting, we did not find differences in the distribution of sleep complaints according the subtype of BD: I, II, and also NOS. Time in bed was longer during depressive episodes than in manic episodes. Nocturnal enuresis occurred more frequently in depressive episode than in manic episodes (see **Table 2**). We found 18 youth BP patients with nocturnal enuresis. We compared the presence of nocturnal enuresis when these patients were in depressive and mania episode and we found that the presence was higher in patients when they were in depressive episode (Qui Square test, p < 0.001). Also, we observed the presence of nocturnal enuresis was higher in prepubertal children compared the adolescent group (Qui Square test, p = 0.009).

**Table 1:** Demographic and clinical characteristics of total sample of children and adolescents with bipolar disorder and sleep complaints (n = 72).^a^

Clinical variables	%
Children n = 29	10 ± 3.0
Adolescents n = 43	15 ± 2.4
Age of 1^st^ episode (y.o.)	7.0 ± 3.4
Gender (Male)	73.6
Family History of PD	97
School impairment	41
Past psychiatric history	18
Type of BD	
BD type 1	80
BD type 2	7
BD type–NOS	13

a y.o., years old; PD, psychiatric disorder; BD, bipolar disorder; BD type–NOS, Bipolar Disorder Not Otherwise Specified.

**Table 2:** Comparison of sleep complaints during depressive episode and manic episode in children and adolescents with bipolar disorder (n = 72).^a^

Sleep complaints	Depressive episode	Manic episode	p value
**Clinical variable**	**%**	**%**	
**Sleep structure**	80.2	78.6	0.812
Initial insomnia	44.4	38.9	0.503
Hypersomnia	37.5	45.8	0.312
Terminal insomnia	79.1	72.2	0.334
Decreased need for sleep	16.1	66.7	<0.001^*^
Night awakening	44.4	44.4	1
Reverse night with day	3.6	1.4	0.002
Restless sleep	75.0	63.8	0.145^*^
Nocturnal enuresis	22.2	1.4	<0.001^b,**^
**Restless sleep**			
Sudden limb movements	56.9	65.2	0.307
Repetitive limb movements	29.1	36.1	0.370
Restless sudden movements	69.4	72.2	0.711
Sleepwalking	20.8	15.3	0.391
Talk during sleeping	51.4	50.0	0.867
Teeth grinding	36.1	37.5	0.862
Night terrors	34.7	33.3	0.860
Nightmares	43.1	44.4	0.875
Recurrent isolated sleep paralysis	15.3	15.3	1

^a,*^Sleep complaints were obtained by psychiatric clinical interview using structure scales to analyze sleep patterns comparing depressive and mania episodes. ns, non significant.

^b,**^Qui Square test, p < 0.01.

Also, daytime energy after a sleep disturbance was higher during both episodes (two tailed test, p < 0.01). We found no association between the medication action and the complaints of nocturnal enuresis in our patients. There was only difference between prepubertal and adolescent group in the unrested sleep that was higher in adolescents in both episodes, and particularly significant during manic episodes (two tailed test, p = 0.048). We did not find difference between genders. Interesting, we found an increase in the reverse night with day during depressive episode, and a higher expression in the decreased need for sleep need parameter in mania episode (see **Table 2**).

## Discussion

This is the first study to analyze the sleep complaints in two phases of BD in children and adolescents. We found differences in each patient, according to the nature of the episode (manic or depressive). Our patients also showed a high level of sleep complaints during manic and depressive episodes. Interestingly, the minority of patients had sleep complaints in both episodes. This finding may be associated with a parental misperception.

Sleep disturbance is often misdiagnosed and unsuspected in adults with refractory depression (14). The refractory depression can be found due to resistance to the treatment, and due to non-adherence to treatment, also a failure to detect an underlying medical comorbidity (15). The decreasing sleep duration, later sleep timing preference, longer sleep latency, increasing nighttime awakenings, and greater sleepiness over follow-up were associated with increasing severity the five psychiatric symptom outcomes over follow-up mania, depression, mood lability, anxiety, inattention/externalizing) (16). Moreover, the manifestations of activity patterns outside of acute episodes add to the accumulating evidence that dysregulation of patterns of activity may constitute a potential biomarker for BD (17). We hypothesized that the presence of interaction between daytime complaints associated with sleep complaints may increase some symptomatic behaviors in these patients, such as agitation, irritability, or others.

There wasn´t equal distribution between subtypes of bipolar disorder in children, and this fact may influence some symptoms in our sample, however the distribution of sleep complaints according the subtype of BD: I, II, and also NOS wasn´t different. Probably sleep can help more in the follow up, and to be considered a biomarker needs more studies, despite the fact that the sleep alterations frequently appear long before the onset of BD, and appear to be related specifically to the polarity of the index episode. The underlying of neurobiology and genetics of bipolar disorder are limited by a heterogeneous clinical phenotype. We would hypothesize that the symptom of each bipolar endophenotypes might be based on the sensitivity to sleep deprivation. The detection and treatment of sleep alterations in special high risk populations may help achieving an earlier detection of the illness (18). In fact, Hernandez et al. (19) in 2017 showed in an elegant study in a sample of 83 patients that sleep disturbances, severe mood instability, bad temper, anxiety symptoms, and aggression were among the most common signs of psychopathology reported in children diagnosed with BD before puberty.

Sleep loss may be one warning signal that can appear before an episode of depression or mania, and it may contribute to a relapse in BD as a prodromal symptom. There is an increase in sleep complaints in children who have been exposed to tragic stress (20). The prevalence of nocturnal enuresis has been reported to be higher children exposed to acute stress (20). Patients with nocturnal enuresis have been associated with sleep instability (21), and it may be a sign of vulnerability or depression. The relationship between sleep and nocturnal enuresis in children and adolescents with BD may be a marker of sleep instability in these patients. Also, unaffected child and adolescent offspring of bipolar parents may have a decrease in the need that may represent an endophenotype of BD in youth (22). In same direction, the sleep complaints in our sample were part of the episode, that were included in the bipolar disorder symptoms. The medications may cause nocturnal enuresis, however in our study as sleep complaints were present only during mood episodes and not in the stable mood, and the restless sleep can be biomarker of mood state.

There is a need to understand the longitudinal processes of the mood disorders, particularly in the early diagnosis in youth. The refractory condition is a common complaint, and there is a role of sleep disorders that was described by McCall et al., in 2019 (14). Youth with bipolar I disorder and a comorbid nightmare disorder appear to be at heightened suicide risk (23), and sleep complaints have been associated with suicidal behavior in youth patients with major depression (24). Our study was developed in a longitudinal setting within the same cohort, and the data should be followed by studies with the longitudinal cohort. There were differences in the polysomnogram studies that showed sleep changes in the expression of REM density in pediatric mood disorders versus attention deficit disorder, and this data can help in the differential diagnosis of the youth patients (25, 26). There is need to evaluate the subjective sleep complaints together with objective analyses. Another limitation of our study was that the protocol did not include the score of Bruni scale (13).

In this study, we tried to develop the sleep phenotype in bipolar disorder, and we used binary analyses that can be less “predictive” but more associative of symptoms. However, the relationship between sleep and daytime complaints in children and adolescents with BD is still unclear. In order to detect mood change in BD, may be necessary to repeat them multiple times or even in both episodes (manic and depressive). Further studies are necessaries to understand the implications of these findings.

## Data Availability Statement

The datasets generated for this study are available on request to the corresponding author.

## Ethics Statement

The studies involving human participants were reviewed and approved by University of Sao Paulo. Written informed consent to participate in this study was provided by the participants' legal guardian/next of kin.

## Author Contributions

ML made all data analyses and discussion. MB was the expert in the clinical interview. LF-I designed the project, arranged for data collection and data tabulation. She also reviewed the analysis, results and discussion.

## Funding

FAPESP: 2008/55402-8.

## Conflict of Interest

The authors declare that the research was conducted in the absence of any commercial or financial relationships that could be construed as a potential conflict of interest.
